# Fatal anogenital exenteration of the intestine

**DOI:** 10.1007/s12024-021-00433-6

**Published:** 2021-10-22

**Authors:** Vanessa Preuss, Kirsten Wöllner, Benedikt Vennemann, Armin Fieguth, Lars Hagemeier, Michael Klintschar

**Affiliations:** 1 Institute of Forensic Medicine, Hanover Medical School, Oldenburg Division, Pappelallee 4, 26122 Oldenburg, Germany; 2 Institute of Forensic Medicine, Hanover Medical School, Carl-Neuberg-Straße 1, 30625 Hannover, Germany

**Keywords:** Fisting, Exenteration of the intestine, Sadism, Rape, Sexual homicide

## Abstract

A case of a sadistically motivated homicide with extraordinary injuries is reported. A 32-year-old woman was naked with signs of severe blunt trauma and oral, vaginal and anal penetration. At the crime scene, the intestine lay next to the woman without connection to the body. During the trial before the criminal court, the perpetrator admitted fisting and inserting several objects into the vagina, anus and oral cavity. Moreover, after anal and vaginal insertion of the hands, large parts of the intestine were torn and pulled out through the anus and the vagina. The results of the forensic pathological examination and additional investigation are discussed and compared with the pertinent literature. This extraordinary case of a sadistically motivated homicide ended with a final judgment that is extremely rare in German jurisdiction.

## Introduction

Homicide to satisfy the sexual instinct represents only a small proportion of all homicides and is distinguished by the sexual motivation of the perpetrator. Sexual activities between the victim and perpetrator and the killing are coincident or in close succession and comprise, for example, sexual intercourse, the insertion of objects into a body orifice, the exposure of the genital region and the implementation of (sadistic) perpetrator fantasies. Under German penal law, sexual motivation for homicide is one of several criteria that qualifies killing a person as *“Mord”* (= intentional killing or murder) as opposed to *“Totschlag”* (= manslaughter).

## Case report

**Fig. 1:**
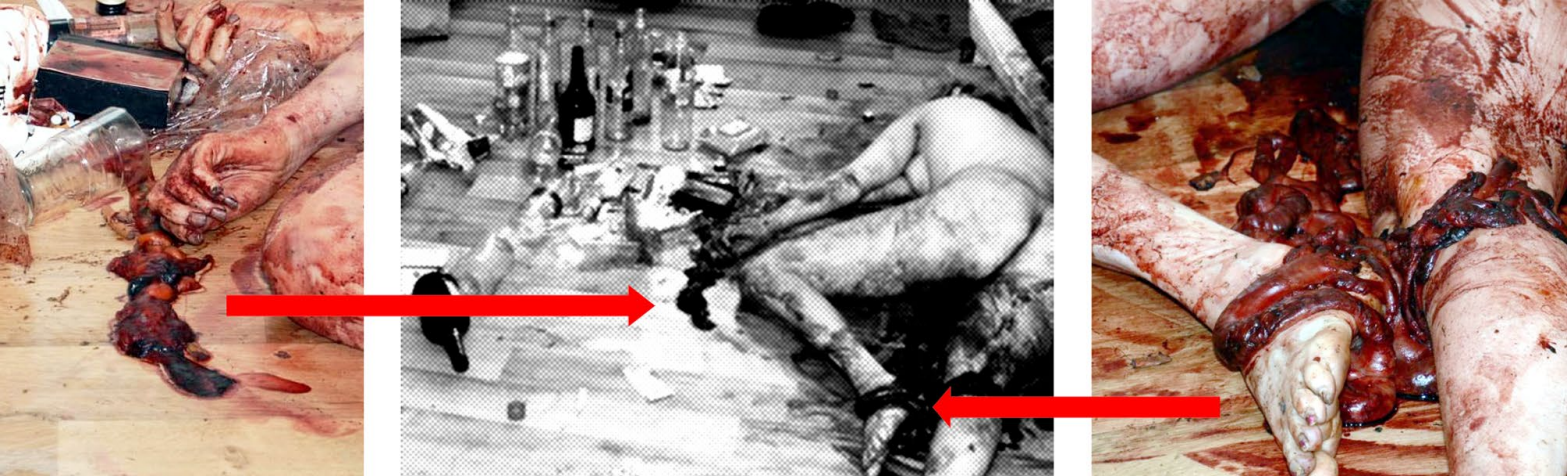
Body with parts of the intestine

In the present case, the 33-year-old perpetrator and the 32-year-old victim lived together. Both were unemployed, addicted to alcohol and known to the police for domestic violence. One morning, the male made an emergency call reporting that he had found his partner dead in the apartment when he woke up. The whole apartment was barely furnished and untidy. The surface of some emptied alcohol bottles was smeared with blood and feces. The deceased was undressed and covered with blood. She was lying in a prone position beside two blood-soaked mattresses on the floor of the living room. In the proximity of the body, several parts of the intestine and pieces of fatty tissue, obviously from the mesentery, were found (Fig. 1). Upon closer examination, there were no signs of sharp violence. Instead, the anus showed deep radial lacerations and hematoma. The vagina seemed to be overstretched and showed lacerations and discharge of blood. Based on these observations, vaginal and anal manipulations and penetrations with perianal exenteration of the intestine were assumed.

## Autopsy findings

### External examination and autopsy

**Fig. 2:**
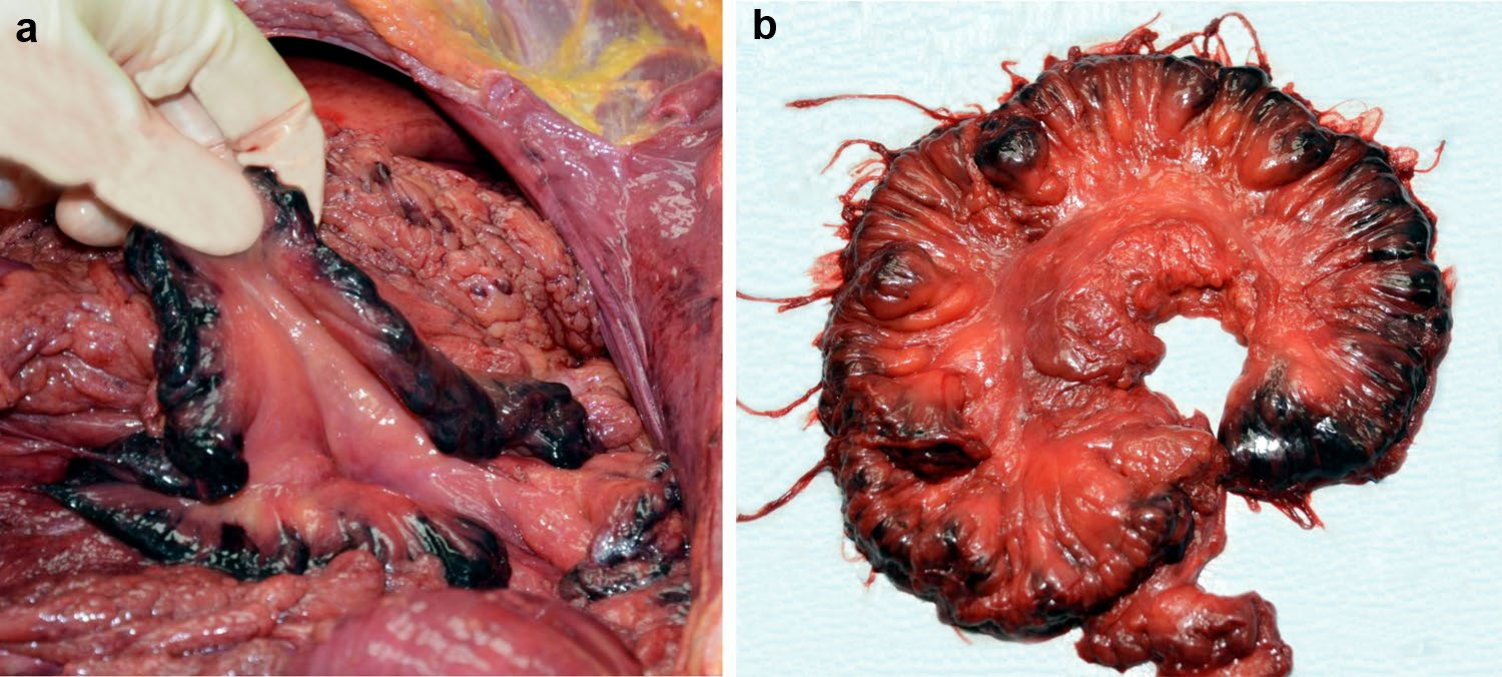
Mesentery with strong bleeding **a** in situ **b** after exenteration during autopsy

The body of the woman had numerous recent hematomas on her face, on the upper and lower extremities and on the back, as well as some older ones; furthermore, cigarette burn lesions of different ages were also observed. Her lips and the oral mucosa were lacerated and discolored, and an incisor was broken. A section of intestine (three by one centimeters) was found on her tongue. The vagina showed many lacerations and hematomas; additionally, the perivaginal fatty tissue had rough disruptions and hemorrhage. The vagina showed an eight centimeter-long laceration at the fundus. The anus showed deep radial tears and the surrounding skin was deeply discolored with hematoma. The rectum and the sigmoid colon were overstretched so that the layers were split over a length of at least 20 cm. In the mucosa of the anus, we found a shard of porcelain. The main finding was a fourfold disruption of the small intestine and the colon with complete interruption of the gastrointestinal passage and detachment of almost the entire small intestine from the mesentery. The tissue of the mesentery showed strong bleeding where the small intestine was removed (Fig. 2a and b). Outside the body, we found a 6.90 m long continuous segment of the small intestine, more than 80 cm of the colon and another piece of small intestine with a length of 22 cm. The wound edges were irregular and frazzled. In addition, as a sign of severe blood loss, the body showed only slight livor mortis, pale organs and subendocardial bleeding in the left ventricle.

### Histological findings

On autopsy, fragments of soft tissue of the vagina, anus and mesentery were saved for histological examination. The samples were stained with hematoxylin–eosin, Masson–Goldner trichrome and Prussian blue stain, and immunohistochemical staining with antibodies against fibronectin and MRP 14 was performed.

**Fig. 3:**
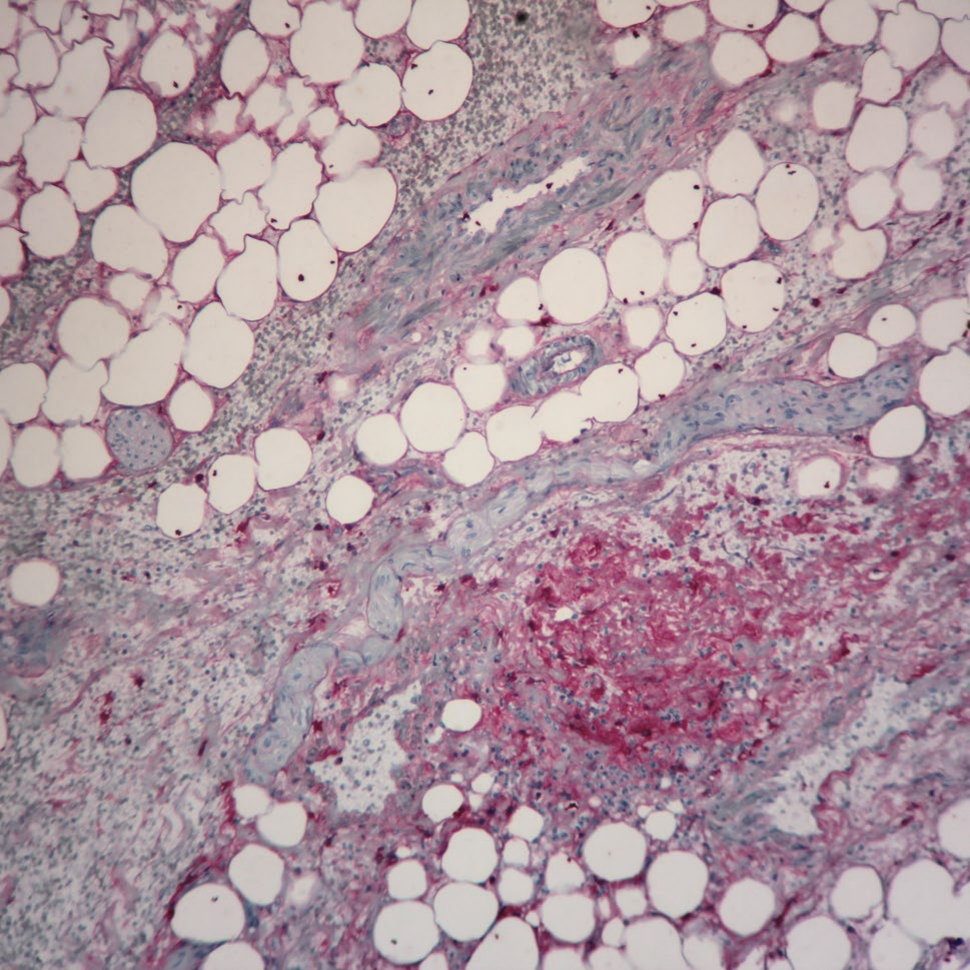
Detection of fibronectin by immunohistochemical staining (× 100)

The strongest bleeding was found in the mesentery, less in the samples of the vagina and anus. The mesentery tissue showed vigorous bleeding and inflammatory cell infiltrates consisting of granulocytes and macrophages as well as fibronectin fiber formation emphasized in the form of foci (Fig. 3). This demonstrates survival for some time after the beginning of the extraction (which was probably a gradual process), even though an exact statement on the survival time is not possible, as the scientific data of immunohistochemical wound reactions refer to skin lesions, not mesenterial tissue. However, when applying these standards, a survival time of at least twenty to thirty minutes can be assumed [1].

### Toxicological findings

The blood alcohol concentration was 4.38 per thousand. Using immunochemical and chromatographic and spectroscopic methods, tetrahydrocannabinol (0.53 ng/ml), 11-OH-THC (0.20 ng/ml), THC-carboxylic acid (16 ng/ml), tranquilizers and opioids were found.

Among these results, the high alcohol level of 4.38 per thousand was in a concentration range in which lethal intoxications are often noted. However, a few weeks before, the woman had an alcohol level of approximately 4.0 per thousand during a police operation without any pertinent deficits.

### Evaluation of the findings

As the cause of death, hemorrhagic shock due to multiple ruptures of the intestine and the mesenteric vessels with perforation of the colon and the vagina caused by manual manipulation (inside the vagina and anus) and the insertion of foreign bodies was established. The piece of porcelain in the anus and the smeared bottles at the crime scene prove the insertion of objects. These findings allow for the conclusion that the intestine was torn out of the abdominal cavity through the ruptured vagina and/or anus. The broken incisor, hemorrhage and overstretching of the lips and oral mucosa indicated additional oral penetration with a fist or objects. The different ages of the hematomas and burns indicated a protracted martyrdom.

### Investigative results and information from the trial

The woman's partner was arrested as a suspect and later indicted at the regional court. The defendant reported recurrent physical violence and sexual abuse of his partner, including fisting with the forearm and the insertion of different subjects, such as glass bottles. Usually, the victim, who suffered from severe alcohol addiction, was heavily intoxicated during these acts. The victim had no masochistic or sadomasochistic preferences.

According to the accused, on the day of the incident, he and his partner consumed alcohol together and initially started “normal” sexual intercourse. It then degenerated into "hard core sex", among other things, with insertion of his fist into the female’s oral cavity and deep fission with his forearm into the vagina and anus. The accused did not comment on tearing out parts of the intestine.

The suspect's exact blood alcohol level at the time of the crime could not be identified. Based on a blood alcohol concentration of 1.52 ‰ the next morning, a theoretical value of up to 5.52 ‰ at the time of crime was calculated.

The psychiatric expert diagnosed alcoholism as well as sexual sadism. From a psychiatric point of view, both diagnoses on their own could have resulted in diminished criminal responsibility (German Criminal Code, § 21).

### Court decision

The perpetrator was sentenced to life imprisonment due to aggravated battery and murder in coincidence with fatal rape, and he was admitted to a hospital for forensic psychiatry. In Germany, such a sentence is usually not given to persons with diminished criminal responsibility. The chamber justified this due to the severe gravity of the deed. The sentence was confirmed by the Federal Court of Justice.

## Discussion

Herein, we report an extraordinarily sadistically motivated homicide with vaginal and anal insertion of at least one hand and tearing out of the intestine through the anus and/or vagina.

The accused showed a pronounced sexual deviation in the sense of sadism. He told the psychiatrist that he felt pleasure and sexual lust in the physical abuse of his partner. In particular, stretching and widening of the body orifices were very satisfying and exciting for him. He inserted his fist up to his wrist into the mouth and throat; moreover, he inserted both fists simultaneously into the vagina as well as into the vagina and anus at the same time and moved them back and forth. Moreover, he penetrated all three body orifices with different objects, such as a bottle of brandy and a spice caster. He enjoyed hard violent sexual intercourse: *“Even if it bled, it was part of it.”[cit.]*

The term *sadism* was introduced by Richard Freiherr von Krafft-Ebing in 1886. It describes the tendency to inflict pain and humiliation on other people and to feel lust [2] and sexual pleasure produced by acts of cruelty and bodily punishment [3]. According to Nitschke [4], the term *sexual sadism* was defined in Krafft-Ebing's work *Psychopathia sexualis* as a feeling of sexual arousal up to orgasm triggered by the urge to humiliate people or inflict pain or wounds on them. In the present case, the psychiatrist based his diagnosis on different screening devices, such as the “Severe Sexual Sadism Scale” [3, 5], which shows strong criterion validity for the diagnosis of sexual sadism disorder. Nine parameters (of 11) of the SSSS were existent. Sexual sadism is recognized as a medical diagnosis (ICD-10 F65.52, DSM-5 302.84 [6, 7]) and should not be confused with the term BDSM (bondage and discipline, dominance and submission, sadism and masochism), which is a widely accepted variety of sexual behaviors [8].

However, sexually motivated sadism can lead to (severe) sexual offenses, up to homicides, as in our case. Generally, homicides related to sex offenses represent only a very small part of all homicides. According to the police crime statistics of the German Federal Office of Criminal Investigation, five sexually motivated homicides were registered in Germany in 2018; this correlates to 1.3% of homicides [9]. Herrmann [10] examined homicides in Hamburg in two periods (1984 to 1989 and 1995 to 2000) and found a sexual motive in 3.2% and 0.9%, respectively. Among sexually motivated homicides in Berlin from 1990 to 2010 [11], only 0.1% were found to be sexually motivated. Moreover, 71% of the sexually motivated homicides were committed due to fear of discovery of the sexual offense [12]. In contrast, killing for the pure gain of sexual pleasure, as can be assumed in the presented case, is rare and should be assigned to a small group of sadists [12].

There are several case reports in the literature that broach the issue of penetration injuries of the vagina or anus by inserting the hand (“fisting”) or objects [13–27]. Sporadic deaths after fisting have also been described [28–30], but in those cases, injuries to vessels, such as the uterine artery, with consecutive blood loss [31] or peritonitis/sepsis after traumatic injury to the colon [29] have occurred. Such extreme violence as in our case, with tearing out almost the entire small intestine and large parts of the colon (including those parts located retroperitoneally) during fisting, is extremely unusual. Thus, to our knowledge, there are only two known cases dealing with perianal exenteration of intestinal parts, both of them in Germany.

Fröb and Püschel [32] describe a sexually motivated homicide in a 66-year-old woman who was found to be seriously injured, covered in blood and partially undressed in her apartment. Next to the body, multiple torn sections of the small and large intestine and fatty tissue were spread, and a section of the small intestine was in a kind of loop around her neck, impressing strangulation. The woman died shortly afterward in a hospital. During the autopsy, vaginal trauma and gashing lacerations in the anus with fresh bleeding were observed, so vaginal manipulation and anal fisting were assumed. The cause of death was hemorrhagic shock because of abdominal trauma.

The second case, which is not published, was examined in our own department. A 45-year-old man was found dead in a male dormitory. A long section of the small intestine stuck out of the anus. In addition, there were other pieces of intestine on the body, which were mostly separated from the mesentery by sharp force. The body also showed signs of blunt violence against the head, multiple cuts in the back and signs of strangulation. However, a sexual motive could not be identified during the trial.

The case presented herein was not only extraordinary and cruel but also a challenge for all involved persons. However, due to interdisciplinary cooperation, an exceptional verdict that stood up in the federal court could be obtained.

## Key points

1. Sadistically motivated homicide to satisfy the sexual instinct represents only a small proportion of all homicides.
2. Sexual sadists inflict physical or psychological suffering on another person to achieve sexual arousal and orgasm.
3. Fatal anogenital exenteration of the intestine is extremely uncommon.
4. In Germany, a life sentence in persons with diminished criminal responsibility is very rare.
5. Due to the severe gravity of the deed, an exceptional verdict was obtained.
